# Selenium Electrophilic Center Responsive to Biological Electron Donors for Efficient Chemotherapy

**DOI:** 10.1002/advs.202412062

**Published:** 2025-02-14

**Authors:** Xiaoyu Qin, Junxian Guo, Hui Li, Hanlong He, Fei Cai, Xinyan Chen, Mingkai Chen, Tianfeng Chen, Li Ma

**Affiliations:** ^1^ Department of Pharmacy and General Surgery of Puning People's Hospital (Guangdong Postdoctoral Innovation Practice Base of Jinan University), College of Chemistry and Materials Science, State Key Laboratory of Bioactive Molecules and Druggability Assessment, MOE Key Laboratory of Tumor Molecular Biology, Guangdong Provincial Key Laboratory of Spine and Spinal Cord Reconstruction Jinan University Guangzhou 510632 China

**Keywords:** biological electron donors, cellular energetic, electrophilic center, selenium atom

## Abstract

Designing drugs to intelligently respond to different ratio of biological electron donors/receptors in cancer cells and normal cells is a promising strategy to achieve highly effective and less toxic chemotherapy. Herein by employing metal center to active the selenium‐containing electrophilic center drug Ru(phtpy‐NO_2_)(phenSe)Cl (**RuSe**) with strongly polarization characteristics are synthesized which can efficiently shuttle electrons from biological electron donors to convert to oxidative stress. The rate of electron transfer at the selenium electrophilic center is 1.81 times higher in cancer cell environments compared to normal cell environments. This results in the selenium electrophilic center being 14.98 times more lethal to cancer cells than to normal cells. Experimental results demonstrate that the transport of electrons process is carried out via selenium radicals intermediate and the rate of electron transport is positively correlated with the polarization properties of the electrophilic center atoms. The selenium electrophilic center transports bioactive electrons to generate a large number of superoxide anions leading to DNA damage and a decrease in mitochondrial membrane potential which further activates the p53 signaling pathway and amplifies the cancer cell‐killing effect after transporting bioactive electrons. This work provides a new avenue for the design of efficient and less toxic chemotherapeutic agents.

## Graphical abstract

Selenium electrophilic center intelligently responds to different ratio of biological electron donors/receptors in cancer cells and normal cells to achieve highly effective and less toxic chemotherapy.

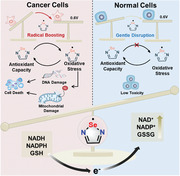

## 1 Introduction

Chemotherapy toxicity is a clinical problem that needs urgent improvement.^[^ ^1^, ^2^, ^3^ ^]^ The characteristics brought about by the unlimited proliferation of tumors provide new opportunities for the design of chemotherapeutic agents.^[^ ^4^, ^5^, ^6^ ^]^ Over the past decades, scientists have conducted in‐depth research on the unique characteristics exhibited by cancer cells, such as deregulating cellular energetics and avoiding immune destruction,^[^ ^7^, ^8^, ^9^, ^10^, ^11^, ^12^, ^13^, ^14^ ^]^ and designed a series of drugs for cancer treatment based on these characteristics. For example, in order to target the characteristics of deregulating cellular energetics, scientists have designed drugs based on glycolysis,^[^ ^15^, ^16^, ^17^, ^18^, ^19^ ^]^ fatty acid metabolism,^[^ ^20^, ^21^, ^22^ ^]^ glutamine metabolism,^[^ ^23^, ^24^ ^]^ the tricarboxylic acid cycle (TCA),^[^ ^25^ ^]^ oxidative phosphorylation (OXPHOS),^[^ ^26^, ^27^ ^]^ and redox metabolism,^[^ ^28^, ^29^ ^]^ demonstrating anticancer effects. The complexity of tumors, however, still calls for more intelligently chemotherapeutic drug design strategies to response tumor characteristics. Herein, we are interested in designing drugs to smart response the different ratio of biological electron donors/acceptors in cancer cells and normal cells to achieve efficient and low‐toxicity chemotherapy.

For biological electron donors, there are three main classes in human body, nicotinamide adenine dinucleotide (NADH), nicotinamide adenine dinucleotide phosphate (NADPH), and glutathione (GSH).^[^ ^30^, ^31^, ^32^ ^]^ By transferring their biological active electrons to substrates to generate their corresponding biological electron acceptors nicotinamide adenine dinucleotide (NAD^+^), nicotinamide adenine dinucleotide phosphate (NADP^+^), and glutathione disulfide (GSSG). Relying on ratio of biological electron donors/acceptors, they regulate the cellular redox environment, cellular energetics, and also a variety of biological processes.^[^ ^33^, ^34^, ^35^ ^]^ Specially, due to the rapid proliferation, the ratio of biological electron donors/receptors in cancer cells and normal cells is different, which providing opportunities to develop strategy to achieve highly effective and less toxic chemotherapy.^[^ ^36^, ^37^, ^38^, ^39^ ^]^ Designing drugs based on the biological electron donors to convert the reduced state to their oxidized state, while converting the antioxidant capacity to oxidative stress, is a promising strategy.^[^ ^40^ ^]^

**Figure 1:**
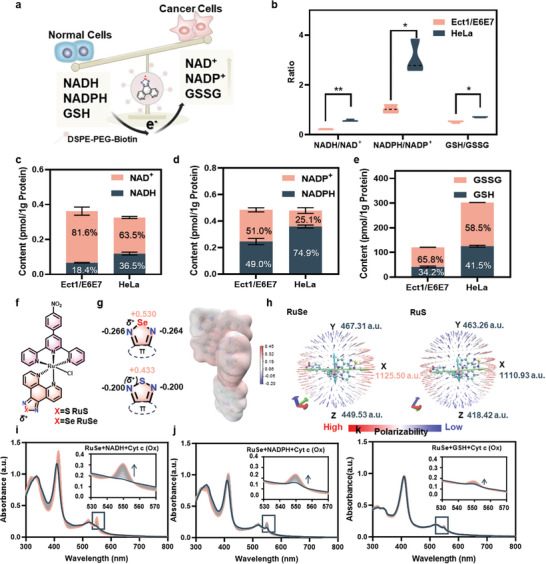
Designing drugs intelligently response to biological electron donor/acceptor ratios to alter intracellular redox balance for chemtherapy. a) Schematic illustration of the strategy to smart response redox balance using electrophilic centers. Ratios b) and contents of NADH/NAD^+^ c), NADPH/NADP^+^ d), and GSH/GSSG e) in cervical cancer cells (HeLa) and cervical normal cells (Ect1/E6E7) (n = 3). f) Chemical structures of **RuS** and **RuSe**. g) Molecular electrostatic potential diagram of **RuSe** and **RuS**, with electronegative regions represented by blue areas and electropositive regions represented by red areas. h) Polarizabilities of RuSe and RuS. i) The changes in the UV–vis spectrum of the reaction between **RuSe** (20 μΜ), NADH (100 μΜ) and Cyt c (Ox) (10 μΜ) for 2 h. j) The changes in the UV–vis spectrum of the reaction between **RuSe** (20 μΜ), NADPH (100 μΜ) and Cyt c(Ox) (10 μΜ) for 2 h. k) The changes in the UV‐visible spectrum of the reaction between **RuSe** (20 μΜ), GSH (100 μΜ) and Cyt c (Ox) (10 μΜ) for 2 h. Data are expressed as mean ± standard deviation; Statistical analysis by unpaired T‐test. ∗*p* < 0.05, ∗∗*p* < 0.01, ∗∗∗*p* < 0.001.

In this work, we are interested in constructing electrophilic centers^[^ ^41^, ^42^ ^]^ to enable the rapid acceptance of electrons from biological electron donors and the subsequent rapid transfer the electrons out. In order to construct such electrophilic centers with “easy‐in, easy‐out” electronic properties, we employed sulfur (S) and selenium (Se) atoms to construct electrophilic centers. Both S and Se have large atomic radii, weak electronegativity and exhibit strong polarizability with a weak ability to hold their outer electrons, espcially Se.^[^ ^43^, ^44^, ^45^ ^]^ By coordination to two high electronegative nitrogen (N) atoms, the ‐N‐Se/S(δ^+^)‐N‐ structure can be used as electrophilic centers to rapid shuttle active electrons,^[^ ^46^, ^47^ ^]^ By employing ruthenium (Ru) metal center to coordinate with the electrophilic center to further active it, the complexes **RuSe** and **RuS** were constructed. Experimental results demonstrated that the synthesized **RuSe** and **RuS** can efficiently shuttle electrons from biological electron donors to convert antioxidant capacity to oxidative stress. By adjusting the ratios of NADH/NAD^+^, NADPH/NADP^+^, and GSH/GSSG, the electrophilic center can intelligently regulate the different systems to be constant at the same potentials. The rate of electron transfer at the selenium electrophilic center is 1.81 times higher in cancer cell environments compared to normal cell environments. And this results in the selenium electrophilic center being 14.98 times more lethal to cancer cells than to normal cells. The transport of electrons process is carried out via Se radicals intermediate, the rate of electron transport is positively correlated with the polarization properties of the electrophilic center atoms. By nanosizing the complexes (**RuSeNPs** and **RuSNPs**) using targeting polymer surfactants, the drugs were better enriched to cancer cells, which further reduced the toxicity to normal cells (**Figure** **1**a). This work provides a new avenue for the design of efficient and less toxic chemotherapeutic agents.

## 2 Results and Discussion

NADH and NADPH are cofactors in many biocatalytic processes, and together with GSH are the major small‐molecule antioxidant system components in cells.^[^ ^48^, ^49^, ^50^ ^]^ All three are biological electron donors and play important roles in the cell, including the maintenance of intracellular redox homeostasis, energy metabolism, and the electron transport chain.^[^ ^51^, ^52^ ^]^ In cancer cells, in order to maintain their high metabolic rate while balancing the excessive oxidative levels, the quantity of their biological electron donors and the ratio of biological electron donors to acceptors differ significantly from those in normal cells.^[^ ^53^, ^54^, ^55^ ^]^ This provides a window for the design of cancer‐specific drugs. Designing drugs that intelligently response to NADH/NAD^+^, NADPH/NADP^+^, and GSH/GSSG ratios to alter intracellular potential levels and ultimately leading to cancer cell death, is a promising pathway. To validate the feasibility of this strategy for cancer therapy, we using NAD^+^/NADH assay kit, NADP^+^/NADPH assay kit, GSH and GSSG assay kits to examine the state of biological electron donors in cervical normal cells (Ect1/E6E7) and cervical cancer cells (HeLa). The results showed that the ratio of biological electron donors/acceptors in HeLa cells were higher than that in normal cells Ect1/E6E7. For the NADH/NAD^+^ ratio, HeLa was 2.53‐fold higher than Ect1/E6E7; for the NADPH/NADP^+^ ratio, HeLa was 2.96‐fold higher than Ect1/E6E7; and for the GSH/GSSG ratio, HeLa was 1.36‐fold higher than Ect1/E6E7 (Figure 1b). Meanwhile, the total contents of biological electron donors (NADPH, NADH, GSH) in cancer cells were higher than those in normal cells, especially GSH and GSSG, which were at a low redox potential (E^θ^_(NADH/NAD+)_ is ‐0.320 V, E^θ^_(NADPH/NADP_^+^_)_ is ‐0.324 V, E^θ^_(GSH/GSSG)_ is _–_ 0.250 V). It's total amount is 2.50 times more than normal cells (Figure 1c–e), this indicates that designing smart‐responsive drugs based on the high biological electron donor/acceptor ratio in cancer cells is a feasible strategy.

To achieve this strategy, we first employed ‐N‐Se/S(δ^+^)‐N‐ electrophilic centers containing ligands, o‐phenanthroline thiadiazole, o‐phenanthroline selenadiazole (phenS, phenSe), to shuttle electrons from biological electron donors, thereby converting antioxidant capacity to oxidize stress. By employing Ru^3+^ coordinated to electrophilic centers as Lewis acid to further active the center, together with auxiliary ligand 4‐(4‐nitrophenyl)‐2,2′:6′,2″‐terpyridine (phtpy‐NO_2_), constructed the complexes Ru(phtpy‐NO_2_)(phenSe)Cl (**RuSe**) and Ru(phtpy‐NO_2_)(phenS)Cl (**RuS**) (Figure 1f). The successful synthesis of the complexes was also demonstrated by mass spectrometry, infrared spectroscopy and ultraviolet‐visible spectrophotometry (Figures S1–S3, Supporting Information), and the purity of the complexes was demonstrated by nuclear magnetic resonance hydrogen spectroscopy (Figure S4,S5, Supporting Information). From Density functional theory (DFT) calculation results we can see, compared to ‐N‐S(δ^+^)‐N‐ electrophilic centers in **RuS**, the ‐N‐Se(δ^+^)‐N‐ in **RuSe** have stronger electrophilic properties (Figure 1g). Meanwhile, the ‐N‐Se/S(δ^+^)‐N‐ electrophilic centers both have large molecular polarizabilities (1125.50 a.u./1110.93 a.u.) (Figure 1h), which suggests that when the reactive electrons are close by, the ‐N‐Se/S(δ^+^)‐N‐ electrophilic centers are able to take the electrons very well and the ‐N‐Se(δ^+^)‐N‐ reactivity is more powerful.

In order to verify whether the designed ‐N‐Se/S(δ^+^)‐N‐ electrophilic center can shuttle the electrons on the biological electron donors well, we used NADH as a representative of the biological electron donors to react with **RuSe**. In order to be able to better observe the occurrence of electron transfer, we chose the electron carrier used in the mitochondrial electron transport chain (ETC): oxidized cytochrome c (Cyt c (Ox)), as a substrate to monitor the reaction of **RuSe** with NADH. Because Cyt c (Ox) is a good electron acceptor, at the same time, the Cyt c (Ox) and reduced cytochrome c (Cyt c (Red)) have characteristic UV absorption peaks at 550 nm, monitoring the changes in the UV peak at 550 nm can be used to detect the progress of the reaction.^[^ ^56^, ^57^ ^]^ We used NADH, NADPH, and GSH as biological electron donors and reacted them with **RuSe**, by using oxidized cytochrome c as substrate we observed that **RuSe** could take up the electrons of NADH, NADPH, and GSH well and deliver them to generate reduced cytochrome c (Figure 1i–k). The above results demonstrate theoretically and practically the feasibility of responding to bioactive electrons via electrophilic centers.

**Figure 2:**
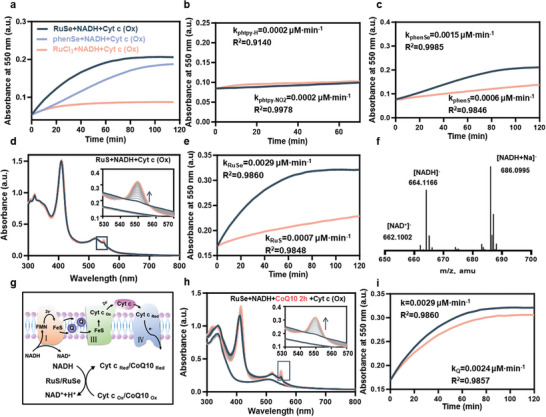
The N‐Se‐N electrophilic center efficiently intercepts electrons from NADH and passes them on to Cyt c. a) The changes in the UV–vis spectrum at 550nm of the reaction between **RuSe** /phenSe/RuCl_3_ (20 μΜ), NADH (100 μΜ) and Cyt c (Ox) (10 μΜ) for 2 h. b) The changes in the UV–vis spectrum at 550nm of the reaction between phtpy‐NO_2_/phtpy‐H (20 μΜ), NADH (100 μΜ) and Cyt c (Ox) (10 μΜ) for 2 h. c) The changes in the UV–vis spectrum at 550nm of the reaction between phenSe/phenS (20 μΜ), NADH (100 μΜ), and Cyt c (Ox) (10 μΜ) for 2 h. d) The changes in the UV–vis spectrum of the reaction between **RuS** (20 μΜ), NADH (100 μΜ) and Cyt c (Ox) (10 μΜ) for 2 h. e) Absorption spectra and rate changes of Cyt c (Red) at 550 nm in Figures d) and Figure 1i). f) High‐resolution liquid chromatography mass spectrometry (negative ion mode) monitoring of **RuSe** (20 µM) + NADH (240 µM) + Cyt c (Ox) (10 µM) at 2 h in deionized water. g) Schematic diagram of the electron transport chain and electrophilic center reaction formula. h) Absorption spectra of **RuSe** (20 μΜ), NADH (100 μΜ) and CoQ10 (100 μΜ) reacted for 2 h, followed by the addition of Cyt c (Ox) (10 μΜ) and continued reaction for 2 h. i) Absorption spectra and rate changes of Cyt c (Red) at 550 nm in Figure d) and h).

In order to further verify whether the electron shuttle process is catalyzed by the ‐N‐Se(δ^+^)‐N‐ electrophilic center or functions through the metal center, we conducted tests under different conditions. The results indicate that the standalone electrophilic center ligand (phenSe) can catalyze the electron transfer from NADH to produce Cyt c (Red), with a catalytic rate slightly lower than that of the electrophilic center activated by a Lewis acid (**RuSe**) (**Figure** **2**a). The standalone metal center Ru^3+^ (Figure 2a) and the auxiliary ligands phtpy‐H/phtpy‐NO_2_ (Figure 2b) show almost no catalytic activity for this process. This process cannot occur in the absence of either the electrophilic center or the biological electron donor (Figure S6, Supporting Information), clearly demonstrating that the transfer of electrons in the biological electron donor is catalyzed by the electrophilic center. The ‐N‐S(δ^+^)‐N‐ electrophilic center has functions similar to that of the ‐N‐Se(δ^+^)‐N‐ in transferring biologically active electrons. Experimental results show that organic ligands with sulfur electrophilic centers (phenS) can also catalyze the transfer of electrons from NADH (Figure 2c). However, the reaction rate of electron transfer from ‐N‐Se(δ^+^)‐N‐ is 250.0% higher than that from ‐N‐S(δ^+^)‐N‐ (Figure 2c). Additionally, after coordination with a Lewis acid (**RuS**), the reaction rate of the ‐N‐S(δ^+^)‐N‐ was enhanced, but its electron transfer rate is lower than that of **RuSe** (Figure 2d). The rate of electron transfer from NADH by **RuSe** is 414.3% higher than that by **RuS** (Figure 2e). Meanwhile, in the mass spectrum we observed that the peak of NAD^+^ at 662.1002 m/z appeared as the reaction proceeded, which also proved the occurrence of the reaction (Figure 2f). In order to verify the specificity of the continued electron transfer after the ‐N‐Se/S(δ+)‐N‐ electrophilic center takes up NADH electrons, we employed another electron‐bearing substrate in the ETC: oxidized coenzyme Q (CoQ) (Figure 2g). Since neither the oxidized nor the reduced state of CoQ has no characteristic absorption peaks. To facilitate monitoring, we added oxidized coenzyme Q (100 µM) to Cyt c (Ox) (10 µM), **RuSe** (20 µM), and NADH (100 µM) as a competitive substrate. The results showed that there was still Cyt c (Red) production in the presence of CoQ (Figure 2h), but the rate of Cyt c (Red) production decreased by 83% and the yield decreased by 95.3% (Figure 2i). This indicates that the oxidized CoQ can also receive electrons shuttled from NADH by the selenium electrophilic center, but the efficiency is lower than that of Cyt c (Ox). The above results collectively demonstrate that the strategy of constructing electrophilic centers using atoms with strong polarizability characteristics for transferring electrons from biologically electron donors is feasible, and the electron transfer rate is proportional to the polarizability of the atoms.

**Figure 3:**
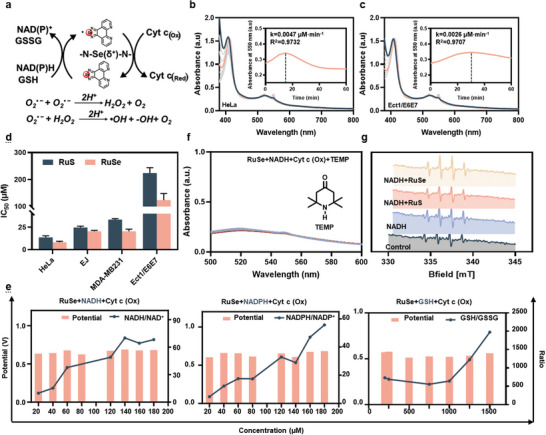
Selenium electrophilic center undergoes radical processes to intelligently respond to biological electron donors. a) Schematic diagram of the free radical production process. b) The changes in the UV‐visible spectrum of the reaction between **RuSe** (20.0 μΜ), NADH (35.6 μΜ), NADPH (109.4 μΜ), GSH (37.6 mΜ), and Cyt c (Ox) (250.0 μΜ) for 1 h. c) The changes in the UV–vis spectrum of the reaction between **RuSe** (20.0 μΜ), NADH (20.0 μΜ), NADPH (69.8 μΜ), GSH (12.4 mΜ) and Cyt c (Ox) (250.0 μΜ) for 1 h. d) Antitumor activity of RuSe and RuS in different cells at 72 h (n = 3). e) Potential and cofactor changes after reaction of **RuSe** with NADH/NADPH/GSH, Cyt c. f) The changes in the UV–vis spectrum of the reaction between **RuSe** (20 µM) + NADH (100 µM)+Cyt c (Ox) (10 µM) + TEMP (5 µM) for 2 h. g) EPR spectral analysis of **RuSe** (50 µM) + NADH (100 µM) + DMPO (5 µM) after interaction.

To further confirm the feasibility and detailed mechanism of the strategy for designing drugs that intelligently respond to different ratios of biologically electron donors in normal and cancer cells (**Figure** **3**a), we first conducted simulation experiments using different concentrations of biologically electron donors measured in normal and cancer cells (Figure 1b–e). By using the mixture of NADH (100 µM for Ect1/E6E7; 100 µM for HeLa), NADPH (349.14 µM for Ect1/E6E7; 309.27 µM for HeLa), and GSH (61.87 µM for Ect1/E6E7; 105.50 µM for HeLa), we measured the reaction catalyzed by **RuSe**. The results showed that although selenium electrophilic centers were able to shuttle electrons under both conditions simulating the cancer cells (Figure 3b) and those simulating normal cells (Figure 3c), but the rate of electron transport by selenium electrophilic centers in the cancer cell environment was 1.81 times higher than that in the normal cell environment. By using the 3‐(4,5)‐dimethylthiahiazo (‐z‐y1)‐3,5‐di‐ phenytetrazoliumromide (MTT) method, we verified the toxicity of complexes on cells, and the results showed that the toxicity of **RuSe** to cancer cells was 14.98 times higher than that of normal cells (Figure 3d), and the toxicity of **RuS** to cancer cells and normal cells showed nearly the same trend with **RuSe**. Moreover, **RuSe** showed better anticancer activity compared to **RuS** (IC_50_ for HeLa: 13.84 µM for **RuS**; 9.36 µM for **RuSe**) and higher safety (SI: 14.90 µM for **RuS**; 15.91 µM for **RuSe**). We found that the anticancer activity of **RuSe** was unaltered under hypoxia (0.1% O_2_) and normal conditions (21% O_2_), suggesting that our strategy is not affected by hypoxia (Figure S7, Supporting Information). Together, these results demonstrate that the strategy of using electrophilic centers with strong polarization properties to intelligently respond biological electron donors is a promising way to develop highly efficient and low‐toxic drugs.

In order to gain a deeper understanding of the electron shuttle process of biologically electron donors, we conducted a mechanistic investigation. By adjusting different concentrations of the biological electron donors, we explored the electron shuttle properties of the electrophilic center. Based on the intensity of the characteristic absorption peak of the product Cyt c (Red) at 550 nm, the concentration of the product was calculated using Beer‐Lambert Law. Furthermore, according to the reaction Equations (1)–(3) involving three different biologically active electron donors catalyzed by the electrophilic center to reduce Cyt c (Ox), the extent of the reaction was inferred (Figure 3e). We found that the ratios of biologically electron donors/receptors after the reaction varied with different initial concentrations of the electron donors, showing a positive correlation with the initial concentration. Based on the analysis using the Nernst equation, it was found that the reaction potential was intelligently regulated and ultimately stabilized around 0.6V by adjusting the ratio of the biologically active electron donors/receptors at different initial concentrations (Figure 3e). This modulation may be one of the reasons for the differing toxicity of the selenium electrophilic center in cancer and normal cells.

(1) $$\mathrm{NADH}+2\mathrm{Cyt}\;c\;(\mathrm{Ox})\;⇌H^{+}+{\mathrm{NAD}}^{+}+2\mathrm{Cyt}\;c\;(\mathrm{Red})\;{rmE}^{⊖}=0.574\;V$$

(2) $$\mathrm{NADPH}+2\mathrm{Cyt}\;c\;(\mathrm{Ox})\;⇌H^{+}+{\mathrm{NADP}}^{+}+2\mathrm{Cyt}\;c\;(\mathrm{Red})\;E^{⊖}=0.578\;V$$

(3) $$2\mathrm{GSH}+2\mathrm{Cyt}\;c\;(\mathrm{Ox})⇌\mathrm{GSSG}+2H^{+}+2\mathrm{Cyt}\;c\;(\mathrm{Red})\;E^{⊖}=0.504\;V$$

To further understand the mechanism of the electron shuttling process, a free radical scavenger, 2,2,6,6‐tetramethylpiperidine‐1‐oxyl radical (TEMP), was added to the mixture of NADH, Cyt c (Ox) and **RuSe** to investigate whether the process involves free radical intermediates. The experimental results showed that after the addition of TEMP, the absorption peak of Cyt c (Red), at 550 nm, essentially disappeared, indicating that the process may undergo a free radical process (Figure 3f). To further confirm the presence of free radicals, we mixed NADH with the selenium electrophilic center and added another free radical trap, 5,5‐dimethyl‐1‐pyrroline‐N‐oxide (DMPO), in the presence of oxygen but without the substrate Cyt c(Ox), to capture the products formed after the reception of electrons by the free radicals from oxygen. Through electron paramagnetic resonance (EPR) detection, we observed the generation of hydroxyl radicals (Figure 3g), which may occur due to the protonation of superoxide anions formed when oxygen receives electrons from selenium radicals, leading to the rapid production of hydrogen peroxide.^[^ ^58^, ^59^ ^]^ Hydrogen peroxide can then couple with superoxide anions to generate hydroxyl radicals (Figure 3a). The above experimental results demonstrate that the selenium electrophilic center undergoes radical processes to intelligently respond to different concentrations of biological electron donors in cancer and normal cells, making it a potential candidate for an efficient and low‐toxicity chemotherapeutic agent.

**Figure 4:**
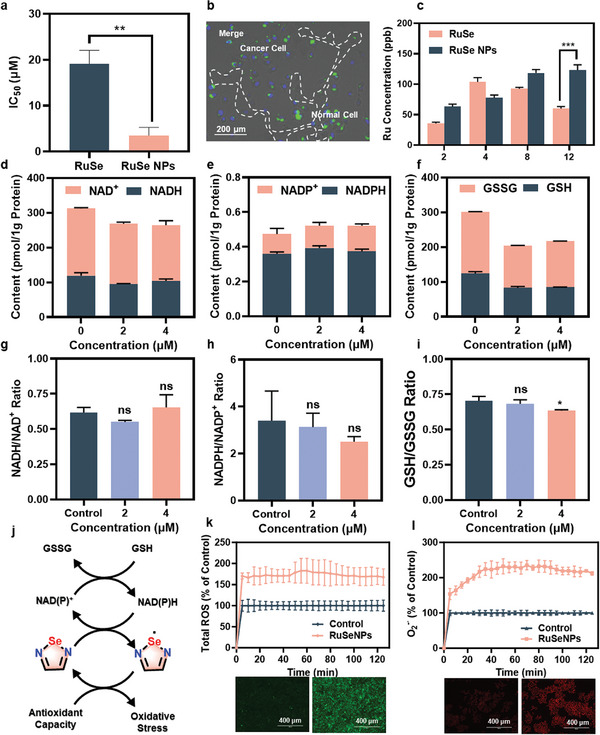
**RuSeNPs** shuttle bioactive electrons to generate superoxide anions. a) Anti‐cancer activity of **RuSeNPs/RuSNPs** in HeLa cells for 48 h (n = 3). b) Co‐culture experiments of cancer cells and normal cells incubated with 4′, 6‐diamidino‐2phenylindole (DAPI) probe in advance, with cancer cell nuclei in blue, fluorescent **RuSeNPs** in green, and normal cells in the dashed portion. c) The changes in drug content in cells after drug treatment for different times (n = 3). d–i) Ratios and contents of NADH, NADPH, and GSH measured after treatment of HeLa cells with **RuSeNPs for 48h** (n = 3). j) The hierarchical relationship diagram of selenium electrophilic centers shuttling electrons from multiple biological electron donors within cells. k‐l) Images of ROS and superoxide anion generation in HeLa cells due to **RuSeNPs**, green, DCF k); red, DHE l). Data are expressed as mean ± standard deviation; Statistical analysis by unpaired T‐test. ∗*p* < 0.05, ∗∗*p* < 0.01, ∗∗∗*p* < 0.001.

To further increase the specificity of drug targeting and in vivo delivery, we employed 1,2‐distearoyl‐sn‐glycero‐3‐phosphoethanolamine‐N‐[biotin(polyethylene glycol)] (DSPE‐PEG‐Biotin) as a surfactant to nanoparticulate **RuSe** (**RuSeNPs**). The biotin group can effectively interact with biotin receptors on the surface of cancer cells, further enhancing the uptake of the drug by cancer cells. The particle size of **RuSeNPs** was roughly 200 nm by dynamic light scattering (DLS) and Transmission Electron Microscopy (TEM) characterization (Figure S8, Supporting Information). Energy dispersive X‐ray spectroscopy (EDS) mapping showed the simultaneous presence of Ru elemental signals, selenium elemental signals, and phosphorus elemental signals (Figure S9, Supporting Information), which proved the successful synthesis of **RuSeNPs**. The results of MTT experiments showed that the nanosized **RuSeNPs** were more cytotoxic to HeLa cell lines compared to **RuSe** (**Figure** **4**a). As shown from the UV–vis spectra (Figure S10, Supporting Information), the **RuSeNPs** also had the ability to shuttle NADH electrons. In addition, co‐culture experiments of cancer cells and normal cells incubated with 4′, 6‐diamidino‐2phenylindole (DAPI) probe in advance revealed (Figure 4b) that **RuSeNPs** with green fluorescence were more likely to be uptake by cancer cells HeLa, and less aggregated in normal cells Ect1/E6E7, with cancer‐cell‐targeting properties. Meanwhile, it was found by cellular uptake assay (Figure 4c) that HeLa cells uptake **RuSeNPs** more than **RuSe**.

In order to gain a deeper understanding of the subsequent effects of drug entry into the cell, after shuttling the bioactive electrons, we performed follow‐up experiments using the HeLa cell line as a representative. First, we examined the changes in the content and ratio of biological electron donors/acceptors after treatment of HeLa cells with **RuSeNPs**. As shown in Figure 4d–i, the NADH/NAD^+^ and NADPH/NADP^+^ ratios did not change significantly after treatment with **RuSeNPs**, but the GSH/GSSG ratio, which has a much lower redox potential, underwent a significant decrease. This may be due to the fact that the standard potential of NADH/NAD^+^, NADPH/NADP^+^ is 0.32 V, while that of GSH/GSSG is 0.25 V. The electrons in NADH and NADPH are compensated from GSH (Figure 4j). The transfer of a large number of bioactive electrons from selenophilic centers in the cell may cause oxidative stress to the cell. By employing 2′,7′‐Dichlorodihydrofluorescein diacetate (DCFH‐DA) and Dihydroethidium (DHE) probes, we examined the intracellular reactive oxygen species (ROS) content, and the results showed that the total ROS content was increased by treatment of the cells with **RuSeNPs** and was mainly manifested in the following ways O_2_^·‐^ level rose (Figures 4k,l; S11, Supporting Information). The above results indicated that **RuSeNPs** robbed intracellular reduced cofactor electrons and then transferred them to O_2_ to generate superoxide anion, which caused oxidative stress.

**Figure 5:**
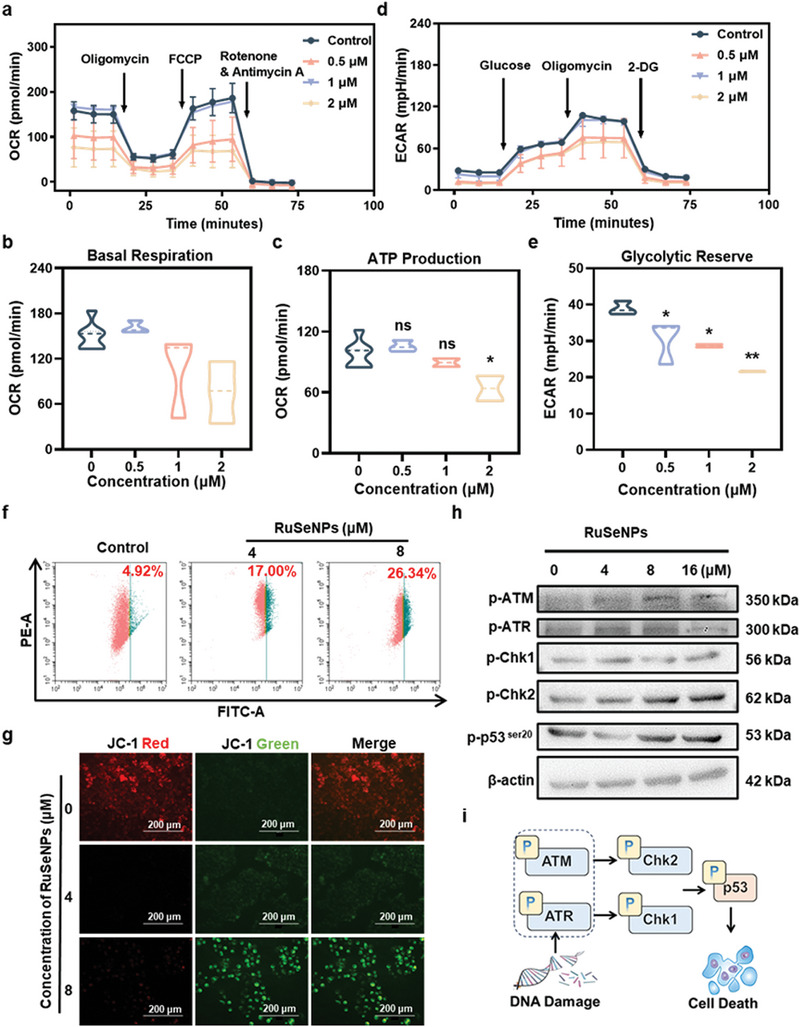
**RuSeNPs** induce DNA damage and mitochondrial destruction thereby promoting cell death. a) Seahorse XF mitochondrial stress test curves after treatment with **RuSeNPs** (n = 3). Key parameters quantified from kinetic characterization of the OCR, including b) basal respiration, c) ATP production (n = 3). d) Seahorse XF glycolytic stress test curves after treatment with different concentrations of **RuSeNPs** (n = 3). e) Glycolytic parameters quantified from kinetic characterization of ECAR (n = 3). f) Changes in mitochondrial membrane potential after treatment of cells with **RuSeNPs**. g) Pictures of mitochondrial membrane potential changes in cells treated with different concentrations of **RuSeNPs**. h) Expression of related proteins in cells treated with **RuSeNPs**. i) Schematic representation of the mechanism by which **RuSeNPs** promotes cell death in vivo. Data are expressed as mean ± standard deviation; Statistical analysis by unpaired T‐test. ∗*p* < 0.05, ∗∗*p* < 0.01, ∗∗∗*p* < 0.001.

Since biological electron donors are major players in glycolysis and mitochondrial respiration processes. To assess the changes in glycolysis and mitochondrial respiratory processes after drug treatment of cells, we monitored the oxygen consumption rate (OCR) and extracellular acidification rate (ECAR) after treatment of HeLa cells with **RuSeNPs** by Agilent‐Seahorse XFe96 analyzer. Basal respiration, oxygen consumption associated with ATP synthesis, maximal respiration, and oxygen consumption associated with proton leakage were determined by sequential addition of mitochondrial respiration regulators (oligomycin, FCCP, and rotenone/antimycin A). The results showed that basal respiration and ATP production were significantly reduced after the treatment of HeLa with different concentrations of **RuSeNPs**, which indicated that the mitochondrial respiration process, especially cellular basal respiration and ATP production, was affected by the treatment of **RuSeNPs** in HeLa cells (**Figure** **5**a–c). Meanwhile, after different concentrations of **RuSeNPs** treated HeLa cells, compared with the control group, from the ECAR results showed (Figure 5d), different concentrations of **RuSeNPs**‐treated HeLa cells at baseline and after stress (addition of oligomycin), the level of ECAR was decreased, along with a significant decrease in glycolytic reserve (Figure 5e). The above results suggest that **RuSeNPs** treatment of cancer cells resulted in a decrease in intracellular glycolysis and mitochondrial respiration levels.

We confirmed the effect of further drug treatment on the subcellular organelle mitochondria after inhibition of mitochondrial respiration. By using a potential‐dependent JC‐1 fluorescent probe, we monitored the changes in mitochondrial membrane potential ΔΨ_m_ after treatment of cells with **RuSeNPs**. As shown in Figure 5f, the JC‐1 probe fluorescence changed from red to green after **RuSeNPs** treatment of cells (Figure 5g), which indicated that the mitochondrial membrane potential decreased and the JC‐1 probe changed from a polymerized state in the mitochondrial matrix to a monomeric state in the cytosol. Meanwhile, it was shown by cell cycle assay that the drug treatment led to cell cycle changes, which caused a rise in Sub‐G1 (Figure S10, Supporting Information). Validation by Western blot assay indicated that **RuSeNPs** mainly up‐regulate phosphorylated ataxia telangiectasia mutated kinase (ATM), ataxia telangiectasia and Rad3‐related kinase (ATR), checkpoint kinase 1 (Chk1), checkpoint kinase 2 (Chk2) and other related proteins to achieve anti‐tumor effects (Figure 5h). The above results suggest that **RuSeNPs** shuttle electrons of biological electron donors causing cellular oxidative stress, decreased mitochondrial membrane potential and DNA damage, which in turn activates the p53 pathway and leads to cell death.

**Figure 6:**
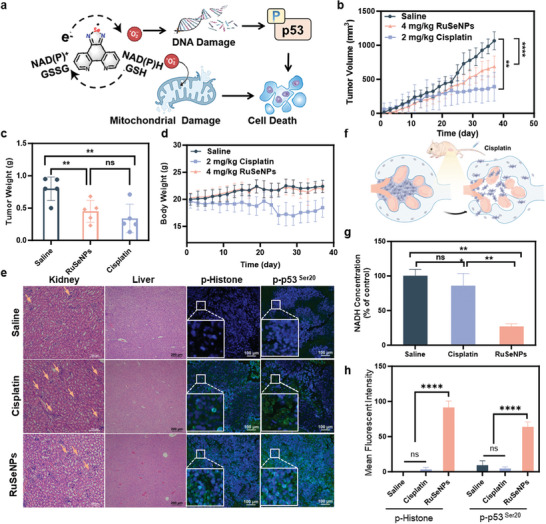
Inhibition of tumor growth in vivo by **RuSeNPs**. a) Schematic diagram of the tumor‐bearing mice model experiment. b) Change in tumor volume after 37 days of treatment with **RuSeNPs** and cisplatin (n = 5). c) Statistical graph of tumor weight in different drug treatment groups (n = 5). d) Body weights of mice treated with **RuSeNPs** and cisplatin for 37 days (n = 5). e) H&E and p‐Histone and p‐p53 staining images in liver and kidney tissues and tumor tissues of mice in different drug treatment groups. f) Schematic diagram of renal tissue damage in mice caused by cisplatin treatment. g) Changes in NADH levels in tumor tissues of mice in different drug treatment groups (n = 3). h) Fluorescence quantitative images of p‐Histone and p‐p53 staining in Figure 6. e) (n = 3). Data are expressed as mean ± standard deviation; Statistical analysis by unpaired T‐test. ∗*p* < 0.05, ∗∗*p* < 0.01, ∗∗∗*p* < 0.001.

Finally, encouraged by the excellent antitumor activity of **RuSeNPs** at the cellular level, we further validated the antitumor effect in vivo (**Figure** **6**a). Animal experiments were performed with a HeLa xenograft nude mouse model by tail vein injection, administered every other day (4 mg/kg). After 38 days of treatment, tumor volume and tumor weight (Figure 6b,c), were suppressed. The inhibitory effect was slightly lower than that of cisplatin at 38 days, but as seen from the mouse body weights (Figure 6d), cisplatin treatment made the mice lose weight significantly with significant toxic side effects, but **RuSeNPs** treatment did not make the mice lose weight significantly with little toxic side effects. Acute kidney injury was the most clinically reported common adverse effect of the highest incidence of cisplatin. H&E staining of liver and kidney tissues showed no significant pathological changes with **RuSeNPs** treatment (Figure 6e). After cisplatin treatment, some of the renal vesicles in mouse kidney tissues were damaged, leading to cellular efflux (Figure 6e,f). Changes in NADH content in tumor tissues after long‐term treatment with the drug were determined by extracting cells from fresh tumor tissues by NAD^+^/NADH assay kit. As shown in Figure 6g, **RuSeNPs** treatment resulted in a significant reduction of NADH content in tumor tissues, and with cisplatin treatment, the change in NADH content was not significant. The changes of p‐Histone and p‐p53 (Ser20) in the tumor sites of mice were determined by immunofluorescence experiments, and the results showed that the expression of p‐Histone and p‐p53 was significantly increased after treatment with **RuSeNPs** (Figure 6h). In In vivo experiments, it was further verified that **RuSeNPs** caused oxidative stress by taking up reducing bioactive electrons and delivering them to substrates such as oxygen, leading to DNA damage and activation of the p53 signaling pathway to induce apoptosis, and the feasibility of the ‐N‐Se/S(δ^+^)‐N‐philotropic center responding intelligently to the biological electron donors to achieve highly efficient and low‐toxicity killing of tumors was also verified.

## 3 Conclusion

Overall, based on the difference in the state of biological electron donors in cancer cells and normal cells, we developed an effective strategy to efficiently and low‐toxicity kill tumors based on the electrophilic centers constructed from elements with strong polarization properties that intelligently respond to the biological electron donors. In this work, the sulfur‐constructed electrophilic centers constructed the complexes **RuSe** and **RuS** by coordination with metal centers, which act as Lewis acids to further activate the electrophilic centers. The selenophilic center shuttle electrons from biological electron donors to generate selenium radicals, then transferring electrons to substrates such as oxygen, converting antioxidant capacity in the cell to oxidative stress. The reaction rate of this process increases with the polarization capacity of the electrophilic center element, and the reaction rate of the selenophilic center transferring electrons from NADH is 4.14 times higher than that of the sulfur electrophilic center. At the same time, the process increases with the increase in the ratio of the biological electron donors/acceptors, and the reaction rate at the ratio of the reduced state in simulated cancer cells is 1.81 times higher than the reaction rate at the ratio of the reduced state in normal cells. After nanosized by surfactants containing cancer‐targeting groups, **RuSeNPs** enhance the selective uptake by cancer cells while the selenophilic center still maintains its reactivity, and the killing effect of **RuSeNPs** on cancer cells is 14.98 times higher than that of normal cells. It was demonstrated that **RuSeNPs** generated a large number of superoxide anions after intercepting and translocating biological electron donors electrons in cancer cells, causing DNA damage and mitochondrial membrane potential decrease, which in turn activated the p53 signaling pathway to induce apoptosis. This work provides a new path for the design of efficient and low‐toxicity chemotherapeutic drugs.

## 4 Experimental Section

### Materials

Chloroform, cytochrome c (Cyt c) were purchased from Shanghai Aladdin Biotechnology Co., ruthenium trichloride was purchased from Bide Pharmaceutical Co., reduced nicotinamide adenine dinucleotide (NADH), reduced nicotinamide adenine dinucleotide phosphate (NADPH), coenzyme Q10 (CoQ10), hydroxyphenyl fluorescein (HPF) were purchased from GLPBIO, Inc., 5,5‐Dimethyl‐1‐pyrroline‐N‐oxide (DMPO) was purchased from Dongren Chemical Technology (Shanghai) Co. Ltd. and reduced glutathione (GSH) was purchased from Guangzhou Scarlett Biotechnology Co., DCFH‐DA probe, dihydroethidium (DHE) were purchased from Sigma., NAD^+^/NADH Detection Kit, NADP^+^/NADPH Detection Kit, GSH and GSSG Detection Kit were purchased from Shanghai Biyuntian Biotechnology Co. Saline was purchased from Beijing Wokai Biotechnology Co.

### Cell Lines

The cell lines used were human cervical cancer cells HeLa cells, human cervical immortalized squamous cells Ect1/E6E7 cells, and all of the above cells were procured from ATCC.

### In Vivo Evaluation of Anti‐Tumor Effects

Human cervical cancer HeLa cells (1×10^6^) were injected into the subcutaneous site of each six‐week‐old female nude mouse to establish a hormonal nude mouse model. After one week, the drug was dissolved in a solution of v(DMF): v(Tween‐80): v(saline) = 2: 10: 88 and administered intravenously at a dose of cisplatin 2 mg/kg, RuSeNPs 4 mg/kg every other day for 37 days, while the control mice received only an equal volume of saline and were monitored on alternate days for body weight and tumor Volume. At the end of the experiment, tumors were exercised, photographed and weighed. Tumor dimensions measured using vernier calipers were used to calculate the volume using the following formula: volume = l × w × w/2, where l was the maximum length and w was the width. All animal experiments were approved by the Ethics Committee for Animal Experiments (IACUC‐20240220‐24).

### Statistical Analysis

All data were expressed as mean ± standard deviation (SD). Differences between experimental groups were analyzed by T‐test and one‐way ANOVA. Survival curves were drawn according to the Kaplan‐Meier method and checked by log‐rank test. Statistically significant differences were defined as having p‐values < 0.05 (*), p < 0.01 (**), and p < 0.01 (***). The number of biologically unique samples and statistical tests used in each experiment was specified in the relevant figure legend. All statistical analyses were conducted using GraphPad Prism 9 software.

## Conflict of Interest

The authors declare no conflict of interest.

## Supporting information



Supporting Information

## Data Availability

The data that support the findings of this study are available in the supplementary material of this article.
