# Supplementary material for: Selenium Electrophilic Center Responsive to Biological Electron Donors for Efficient Chemotherapy
Source: Adv Sci (Weinh). 2025 Feb 14;12(14):2412062. doi: 10.1002/advs.202412062 (PMC11984860; doi:10.1002/advs.202412062)
Supplement: Supplementary file 1 — Supporting Information [file ADVS-12-2412062-s001.docx]

Supporting Information

**Selenium Electrophilic Center Responsive to Biological Electron Donors for Efficient Chemotherapy**

Xiaoyu Qin,^‡^ Junxian Guo,^‡^ Hui Li,^‡^ Hanlong He, Fei Cai, Xinyan Chen, Mingkai Chen, Tianfeng Chen,* and Li Ma,*

Department of Radiation Oncology of Puning People’s Hospital, Department of Chemistry of Jinan University, Key Laboratory for Regenerative Medicine of Ministry of Education, Guangdong Provincial Key Laboratory of Spine and Spinal Cord Reconstruction, Jinan University, Guangzhou, 510632, China.

E-mail: chem_mali@jnu.edu.cn, tchentf@jnu.edu.cn

**Table of Contents**

[1. Experimental Procedures 3](#_Toc172053592)

[1.1 Materials 3](#_Toc172053593)

[1.2 Instrumentation 4](#_Toc172053594)

[1.3 Synthesis 4](#_Toc172053595)

[1.4 Mass spectrometric characterization 6](#_Toc172053596)

[1.5 Infrared characterization 7](#_Toc172053597)

[1.6 Ultraviolet characterization 7](#_Toc172053598)

[1.7 Nuclear magnetic hydrogen spectroscopy characterization 7](#_Toc172053599)

[1.8 Morphological characterization of nanoparticles 7](#_Toc172053600)

[1.9 Cell lines and their culture methods 7](#_Toc172053601)

[1.10 Drug uptake assay by cells 7](#_Toc172053602)

[1.11 In vitro anti-tumor assay 8](#_Toc172053603)

[1.12 Detection of intracellular reactive oxygen species level 8](#_Toc172053604)

[1.13 Caspase activity analysis 9](#_Toc172053605)

[1.14 Determination of cellular respiration 9](#_Toc172053606)

[1.15 Cell cycle assay 9](#_Toc172053607)

[1.16 Mitochondrial membrane potential 10](#_Toc172053609)

[1.17 Protein blotting assay 10](#_Toc172053610)

[1.18 In vivo evaluation of anti-tumor effects 11](#_Toc172053611)

[1.19 Statistical analysis 11](#_Toc172053612)

[2. Results and Discussion 12](#_Toc172053613)

[3. References 16](#_Toc172053621)

**1. Experimental Procedures**

**1.1 Materials**

5,6-diamino-1,10-o-phenanthroline, p-dimethylaminobenzaldehyde, benzaldehyde were purchased from Sahn Chemical Technology (Shanghai) Co., triethylamine, glacial acetic acid were purchased from Tianjin Jindong Tianzheng Fine Chemical Reagent Factory., 2-acetylpyridine, selenium dioxide, 2,2,6,6-tetramethylpiperidine-1-oxyl radical (TEMP) were purchased from Shanghai McLean Biochemical Technology Co.，4-Nitrobenzaldehyde was purchased from Beijing Bailing Wei Technology Co., Ltd, p-methylbenzaldehyde was purchased from Shanghai Adamus Reagent Co., Ltd, and ruthenium trichloride was purchased from Bide Pharmaceutical Co., DSPE-PEG2000-Biotin was purchased from Pengshuo Bio., Reduced nicotinamide adenine dinucleotide (NADH), reduced nicotinamide adenine dinucleotide phosphate (NADPH), coenzyme Q10 (CoQ10), hydroxyphenyl fluorescein (HPF), Inc., 5,5-Dimethyl-1-pyrroline-N-oxide (DMPO) was purchased from Dongren Chemical Technology (Shanghai) Co. Ltd. and reduced glutathione (GSH) was purchased from Guangzhou Scarlett Biotechnology Co., Tetrazolium blue (MTT), propidium iodide (PI), JC-1 probe, DCFH-DA probe, dihydroethidium (DHE), Ac-DEVD-AMC (caspase 3), Ac-IETD-AFC (caspase 8), Ac-LEHD-AFC (caspase 9) were purchased from Sigma., NAD^+^/NADH Detection Kit, NADP^+^/NADPH Detection Kit, GSH and GSSG Detection Kit were purchased from Shanghai Biyuntian Biotechnology Co. Saline was purchased from Beijing Wokai Biotechnology Co. PBS and trypsin were purchased from Guangzhou Jiebes Biological Company.All the chemicals and solvents were analytically pure.

The water used in all experiments was ultrapure by a Milli-Q water purification system from Millipore.

**1.2 Instrumentation**

The complexes were characterized using a 1290LC-6545 QTOF MS high-resolution liquid-mass spectrometer, an AVANCE Ⅲ HD 600MHz nuclear magnetic resonance spectrometer, a Perkin Elmer infrared spectrometer, a Cary5000 UV-visible spectrophotometer, and an LF-1701007 fluorescence spectrometer. The morphology of nanoparticles was characterized using a NANO ZS particle size analyzer, Hitachi H-7650 transmission electron microscope (Hitachi). Absorbance was detected using an ELX800/Cytation 5 multifunctional fluorescence enzyme marker (Bio-Tek, USA). Cells were collected as well as photographed using a CytoFLEXS flow cytometer and EVOS FL Auto fluorescence microscope.

**1.3 Synthesis**

**1.3.1 Synthesis of ligands**

**Synthesis of phenSe ^[1]^**

0.210 g (1 mmol) of 5,6-diamino-1,10-o-phenanthroline was dissolved in 5 mL of glacial acetic acid and 0.110 g (1 mmol) of SeO_2_ was dissolved in 3 mL of distilled water. The two were then poured into a beaker and mixed, and the reaction was stirred at room temperature for 30 min, after which about 7 mL of ammonia was added dropwise to the above mixture, and the solution gradually turned into pink color and a precipitate was formed. The solution was then filtered and washed 2-3 times with distilled water and dried. The solid was redissolved with dichloromethane: methanol (1:1), and then crystallized by natural evaporation to collect pink needle crystals.

**Synthesis of phtpy-NO_2_**

2-Acetylpyridine (2.420 g, 20.0 mmol) and 4-nitrobenzaldehyde (1.420 g, 9.4 mmol) were weighed into a round-bottomed flask and 70 mL of anhydrous ethanol was added. After the two were dissolved, 0.350 g NaOH and 10 mL ammonia (30%) were added to the solution. Then stirred at room temperature for 2 h. After the reaction, the brown precipitate was filtered and dried.

**Synthesis of phtpy**

2-Acetylpyridine (2.420 g, 20.0 mmol) and benzaldehyde (1.000 g, 9.4 mmol) were weighed into a round-bottomed flask and 70 mL of anhydrous ethanol was added. After the two were dissolved, 1.050 g NaOH and 30 mL ammonia (30%) were added to the solution. Then stirred at room temperature for 12 h. After the reaction, filtered to obtain a yellow precipitate, dried.

**1.3.2 Synthesis of Precursor**

**Synthesis of Ru(phtpy-NO_2_)Cl_3_**

Weighed phtpy-NO_2_ (0.354 g, 1 mmol) and RuCl_3_-3H_2_O (0.456 g, 2.2 mmol) were first added to the reaction flask. The air in the reaction flask was withdrawn and N_2_ was passed in. 45 mL of anhydrous ethanol was added and the solution was refluxed overnight under N_2_ to give a reddish brown liquid. The anhydrous ethanol was removed and the solid was filtered, washed with water to remove excess RuCl_3_-3H_2_O and dried to give a brown solid.

**1.3.3 Synthesis of cooperative substance**

**Synthesis of RuSe**

Weighed Ru(phtpy-NO_2_)Cl_3_ (0.561 g, 1 mmol), phenSe (0.286 g, 1 mmol) and a few drops of triethylamine were first added to the reaction flask, the air inside the reaction flask was withdrawn, N_2_ was passed through the flask, and 45 mL of anhydrous ethanol was added under the condition of N_2_. The solution was refluxed at 80 °C under N_2_ atmosphere overnight. After the reaction, the solution was cooled to room temperature, a saturated solution of sodium perchlorate was added, the solvent was removed by filtration and dried to give a brown-red solid. Afterwards, the crude product was purified by silica gel column chromatography using dichloromethane and methanol as eluent to finally obtain a purplish-red solid.

ESI-MS (in MeOH): m/z = 776.9606 [M] ^+^。^1^H NMR (600 MHz, DMSO-*d*_6_) δ 10.36 (dd, *J* = 5.4, 1.4 Hz, 1H), 9.35 (s, 2H), 9.30 (dd, *J* = 8.1, 1.4 Hz, 1H), 9.00 (d, *J* = 8.1 Hz, 2H), 8.76 – 8.70 (m, 1H), 8.66 (d, *J* = 8.3 Hz, 2H), 8.55 (d, *J* = 8.2 Hz, 2H), 8.48 (dd, *J* = 8.1, 5.3 Hz, 1H), 8.06 – 8.01 (m, 2H), 7.90 – 7.81 (m, 1H), 7.70 (dd, *J* = 5.5, 1.4 Hz, 2H), 7.46 (dd, *J* = 7.9, 5.6 Hz, 1H), 7.33 (t, *J* = 6.6 Hz, 2H).

**Synthesis of RuS**

Weighed Ru(phtpy-NO_2_)Cl_3_ (0.561 g, 1 mmol), phenS (0.238 g, 1 mmol) and a few drops of triethylamine were first added to the reaction flask, the air inside the reaction flask was withdrawn, N_2_ was passed through the flask, and 45 mL of anhydrous ethanol was added under the condition of N_2_. The solution was refluxed at 80 °C under N_2_ atmosphere overnight. After the reaction, the solution was cooled to room temperature, a saturated solution of sodium perchlorate was added, the solvent was removed by filtration and dried to give a brown-red solid. After that, the crude product was purified by silica gel column chromatography using dichloromethane and methanol as eluent to finally obtain a purple-red solid.

ESI-MS (in MeOH): m/z = 729.0162 [M] ^+^。^1^H NMR (600 MHz, DMSO-*d*_6_) δ 10.46 – 10.38 (m, 1H), 9.39 (dd, *J* = 8.3, 1.5 Hz, 1H), 9.35 (s, 3H), 9.00 (d, *J* = 8.9 Hz, 2H), 8.65 (d, *J* = 9.1 Hz, 2H), 8.60 – 8.53 (m, 3H), 8.04 (td, *J* = 7.9, 1.6 Hz, 2H), 7.97 – 7.90 (m, 1H), 7.71 – 7.62 (m, 2H), 7.53 (dd, *J* = 8.3, 5.8 Hz, 1H), 7.36 – 7.28 (m, 2H).

**1.3.4 Nanosizing of ruthenium complexes**

The corresponding complexes were weighed and dissolved in DMSO to configure a 10 mM concentration of the reservoir solution. Tween-80 was dissolved in ultrapure water at a concentration of 5 mg/mL, and then DSPE-PEG2000-Biotin was dissolved in a mixture of chloroform and DMSO reservoir solution at a concentration of 1 mg/mL (4:1, v/v), and the volume ratio of the organic phase to water was kept as 1:4, and then the mixture was added into the stirred aqueous solution of Tween-80 at a uniform rate. The organic solvent was allowed to evaporate slowly and stirred for 12 h. A homogeneous and stable purple-red nanosystem was finally obtained. Finally, the raw material and the remaining tiny particles were removed by overnight dialysis using a dialysis bag, and their concentration (calculated as the concentration of the metal center Ru) was determined by inductively coupled plasma mass spectrometry (ICP-MS).

**1.4 Mass spectrometric characterization**

The complex powder to be tested was dissolved in about 1 mL of chromatographic grade methanol, filtered through a 0.22 µm aqueous filter head and loaded into a mass spectrometry vial, tested using the positive ion mode of mass spectrometry in the high-resolution liquid-mass spectrometry (HRL-MS) coupler, and finally analyzed using AgtQual and Isopro software.

**1.5 Infrared characterization**

The IR spectra of the complexes were determined using an IR spectrometer with a scanning range of 4000-400 cm^-1^. After exporting the data, it was analyzed by graphing with Origin 2019b software.

**1.6 Ultraviolet characterization**

The samples were dissolved in methanol solution configured to a concentration of about 50 μM, 2 mL of the liquid was taken into a UV dish, and the UV spectra of all the ligands and complexes were measured using a UV-visible spectrophotometer in the wavelength range of 200-800 nm, and after exporting the data, the data were graphically analyzed using GraphPad software.

**1.7 Nuclear magnetic hydrogen spectroscopy characterization**

A certain mass of the complex powder was weighed and dissolved in 600 μL of deuterated DMSO, which was transferred to a NMR tube and detected using a 600 M NMR spectrometer and finally analyzed using MestReNova software.

**1.8 Morphological characterization of nanoparticles**

The synthesized nanoparticles were characterized by Malvern particle size analyzer to determine their size and their morphology was studied using transmission electron microscopy (TEM).

**1.9 Cell lines and their culture methods**

The cell lines used were human cervical cancer cells HeLa cells, human bladder cancer cells EJ cells, human breast cancer cells MDA-MB-231 cells, human cervical immortalized squamous cells Ect1/E6E7 cells, and all of the above cells were procured from ATCC. The cell experiments were performed with cells in logarithmic growth phase and cultured in DMEM medium containing 1% penicillin-streptomycin and 10% fetal bovine serum at 37 ℃ (5% CO_2_).

**1.10 Drug uptake assay by cells**

HeLa cells in logarithmic growth phase were taken and inoculated in 6 cm dishes at a density of 4×10^5^ cells / well, the cells were cultured overnight to make the cells adherent to the wall, then 6 μM of **RuSe** complexes and **RuSeNPs** were added for incubation at different time points of 2, 4, 8, and 12 h. The cells were washed with PBS for 2-3 times after removal of the medium and then the cells were collected and cell counting was performed. After digestion of the cells in aqua regia, the intracellular content of **RuSe** complex and **RuSeNPs** drug was determined using ICP-MS.

**1.11 In vitro anti-tumor assay**

HeLa cells in logarithmic growth phase were taken and inoculated in 96-well plates at a density of 2×10^3^ cells/well, and the cells were cultured overnight to make the cells adherent to the wall, then different concentrations of Ru complexes and nanosized complexes were added, and incubated for 48 h and 72 h, respectively, and then 30 μL of MTT solution with the concentration of 5 mg/mL was added to each well and incubated for 4 h. After that, the supernatant was aspirated. After that, 150 μL of DMSO was added to each well to fully dissolve. The absorbance value of the solution at 570 nm was determined by using an enzyme labeling instrument, and finally, the IC_50_ of the drug on the cells was calculated.

**1.12 Detection of intracellular reactive oxygen species level**

Determination of total reactive oxygen species level: HeLa cells in logarithmic growth phase were inoculated in 96-well plates at a density of 2×10^4^ cells/well, and the cells were cultured overnight to make the cells adherent to the wall, then the medium was aspirated, and 10 μM DCFH-DA probe diluted in PBS was added, and different concentrations of **RuSeNPs** were added after 30 min incubation. The change of fluorescence intensity at Ex=488 nm and Em=525 nm was measured by Cytation multifunctional fluorescence enzyme marker.

Determination of superoxide anion level: HeLa cells in logarithmic growth phase were inoculated in 96-well plates at a density of 2×10^4^ cells/well, and the cells were cultured overnight to make the cells adherent to the wall, then the medium was aspirated, and 10 μM DHE probe diluted in PBS was added, and **RuSeNPs** were added at different concentrations after incubation for 30 min. The change in fluorescence intensity at Ex=300 nm, Em=610 nm was measured using a Cytation multifunctional fluorescent enzyme marker.

Determination of hydroxyl radicals: HeLa cells in logarithmic growth phase were inoculated in 96-well plates at a density of 2×10^4^ cells/well, and the cells were cultured overnight to allow the cells to adhere to the wall, then the medium was aspirated, and 10 μM HPF probe diluted in PBS was added, and **RuSeNPs** were added at different concentrations after incubation for 30 min. The change of fluorescence intensity at Ex=488 nm and Em=525 nm was measured by Cytation multifunctional fluorescent enzyme marker.

**1.13 Caspase activity analysis**

HeLa cells were inoculated in 10 cm dishes at a density of 10 × 10^5^ cells per well. After cell adhesion, different concentrations of **RuSeNPs** were added. after a total of 48 h of incubation, cellular proteins were collected, and the protein concentration of the cells after the drug was detected by the bovine serum albumin (BSA) method. Then 100 μg of collected proteins and 5 μL of enzyme substrates (Ac-DEVD-AMC for caspase 3, Ac-IETD-AMC for caspase 8, and Ac-LEHD-AMC for caspase 9) were added to each well, and then the solution was replenished to 200 μL with PBS. After incubation for 2 h at 37°C, the assay was performed by a multifunctional fluorescent enzyme marker.

**1.14 Determination of cellular respiration**

2 × 10^5^ HeLa cells/mL were inoculated on XFe96 FluxPax microplates. After treatment with **RuSeNPs** at concentrations of 2 μM, 1 μM, 0.5 μM, and 0.25 μM for 12 h, the medium was replaced with XF basal medium, respectively. OCR was performed using the Seahorse XF Cellular Mitochondrial Stress Kit along with 1.0 μM Oligomycin, 1.5 μM carbonyl cyanide-4 (trifluoromethoxy) phenylhydrazone (FCCP), 0.5 μM Rotenone, and Antimycin A on an XF96 analyzer (Agilent Technologies). ECAR was performed using the Seahorse XF Glycolytic Stress Kit along with 10 mM glucose (Glucose), 0.1 μM oligomycin, and 50 mM 2-deoxyglucose (2-DG) in an XF96 analyzer.

**1.15 Cell cycle assay**

HeLa cells were inoculated in 6-well plates at a density of 8×10^4^ cells/well, and the cells were cultured overnight to make the cells adherent to the wall, then different concentrations of **RuSeNPs** were added, and the cells were collected after incubation for 48 h. The cells were stained with pyridinium iodide (PI), and then detected on a flow cytometer, and the number of cells collected was 10000.The data were processed and analyzed by Modfit software.

**1.16 Mitochondrial membrane potential**

HeLa cells were inoculated in 6-well plates at a density of 8×10^4^ cells/well, and the cells were cultured overnight to allow the cells to adhere to the wall, then different concentrations of **RuSeNPs** were added to be tested, and the JC-1 probe was added after 48 h of incubation for a further 30 min, respectively. The changes in the mitochondrial membrane potential (Δ_Ψm_) were analyzed by flow cytometry.

**1.17 Protein blotting assay**

HeLa cells in logarithmic growth phase were taken and inoculated in 10 cm dishes at a density of 1×10^6^ cells / well, and the cells were incubated overnight to make the cells adherent to the wall, then different concentrations of **RuSeNPs** were added, and the cellular proteins were collected after a total incubation period of 48 h. The cells were incubated with lysis buffer to extract the total cellular proteins. The cells were incubated with lysis buffer and total cellular proteins were extracted. Protein concentration in treated HeLa cells was assayed by the bovine serum albumin (BSA) method. Equal amounts (30 µg) of proteins were electrophoresed in 10% SDS-PAGE polyacrylamide gels, then transferred to polyvinylidene fluoride (PVDF) membranes and blocked for 2 h with 5% skim milk in TBST buffer. The membrane was then incubated with the corresponding primary antibody at a dilution of 1:1000 at 4°C overnight. They were then incubated with secondary antibodies at a dilution of 1:3000 for 2 h at 4°C and washed three times with TBST. Finally, the color was developed with a Kodak X-ray chemiluminescence detector. β-actin was used to confirm equal loading and transfer of proteins. Protein blotting bands were analyzed in grayscale using Image J software.

**1.18 In vivo evaluation of anti-tumor effects**

Human cervical cancer HeLa cells (1×10^6^) suspended in 100 µL PBS were injected into the subcutaneous site of each six-week-old female nude mouse to establish a hormonal nude mouse model. The mice were randomly assigned to three groups of five mice each. After one week, the drug was dissolved in a solution of v(DMF): v(Tween-80): v(saline) = 2: 10: 88 and administered intravenously at a dose of cisplatin 2 mg/kg, **RuSeNPs** 4 mg/kg every other day for 37 days, while the control mice received only an equal volume of saline and were monitored on alternate days for body weight and tumor Volume. At the end of the experiment, tumors were excised, photographed and weighed. Tumor dimensions measured using vernier calipers were used to calculate the volume using the following formula: volume = l × w × w/2, where l is the maximum length and w is the width. All animal experiments were approved by the Ethics Committee for Animal Experiments (IACUC-20240220-24).

**1.19 Statistical analysis**

The experiment was repeated at least three times and data were expressed as mean ± standard deviation. Differences between experimental groups were analyzed by t-test and one-way ANOVA. p-value <0.05 was considered significant.

**2. Results and Discussion**


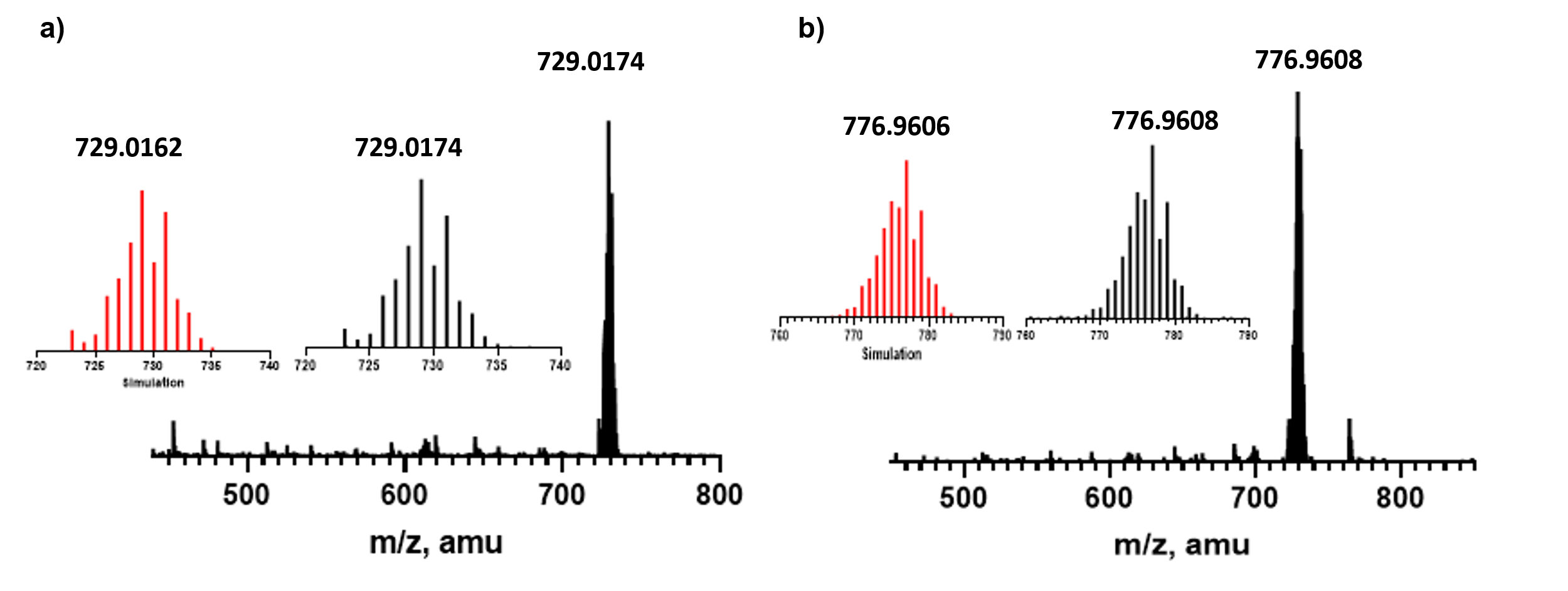


**Figure S1.** Mass spectrometry of a) **RuS** and b) **RuSe** in methanol solution (positive ion mode).


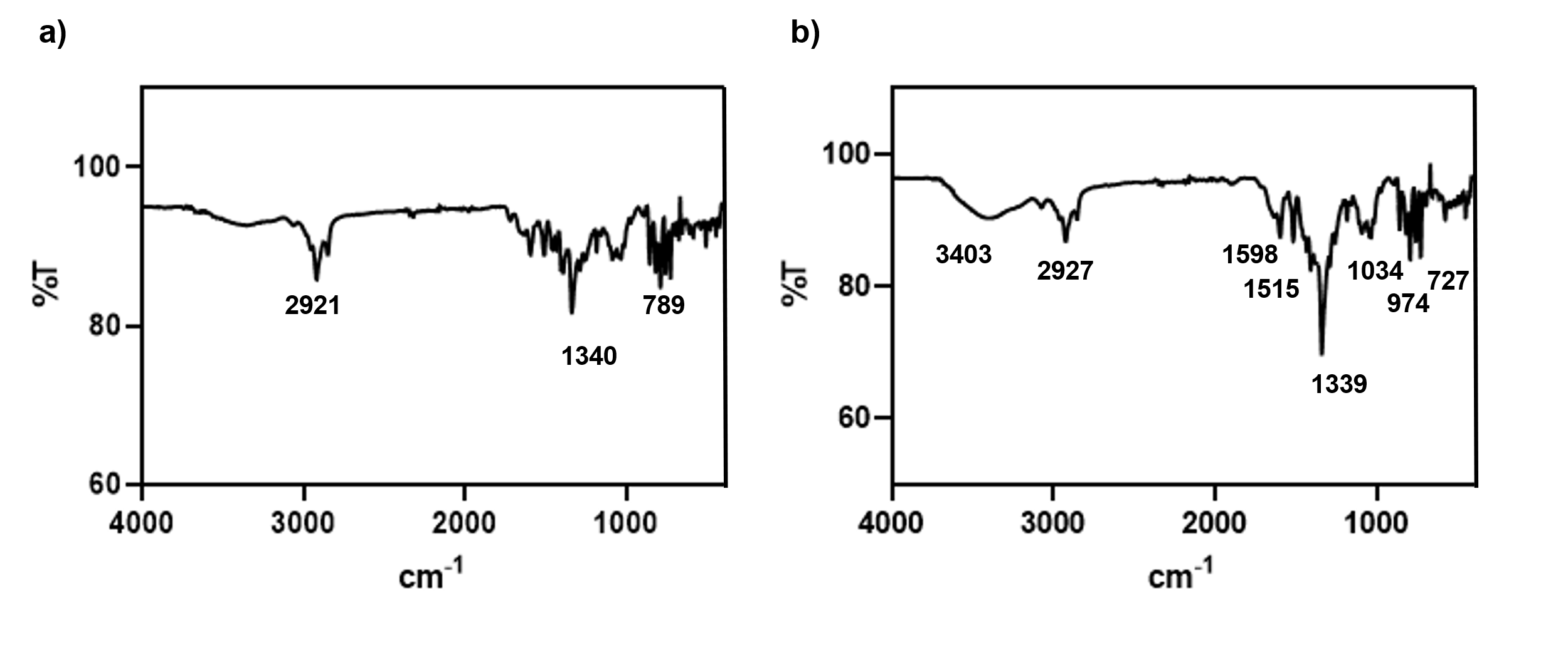


**Figure S2.** The Infrared spectra of a) **RuS** and b) **RuSe**.


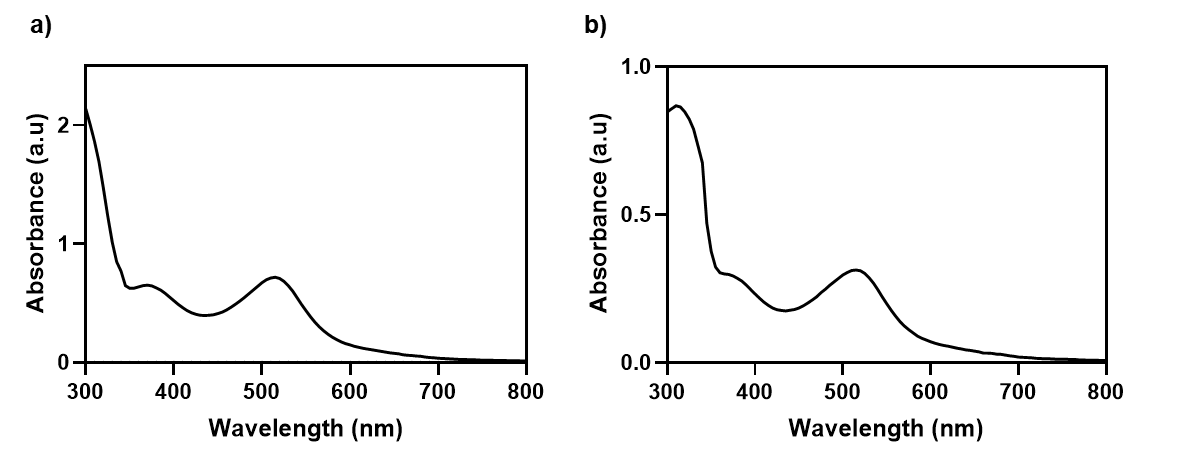


**Figure S3.** UV spectra of the a) **RuS** and b) **RuSe** in methanol solution at a concentration of 50 μM.

**
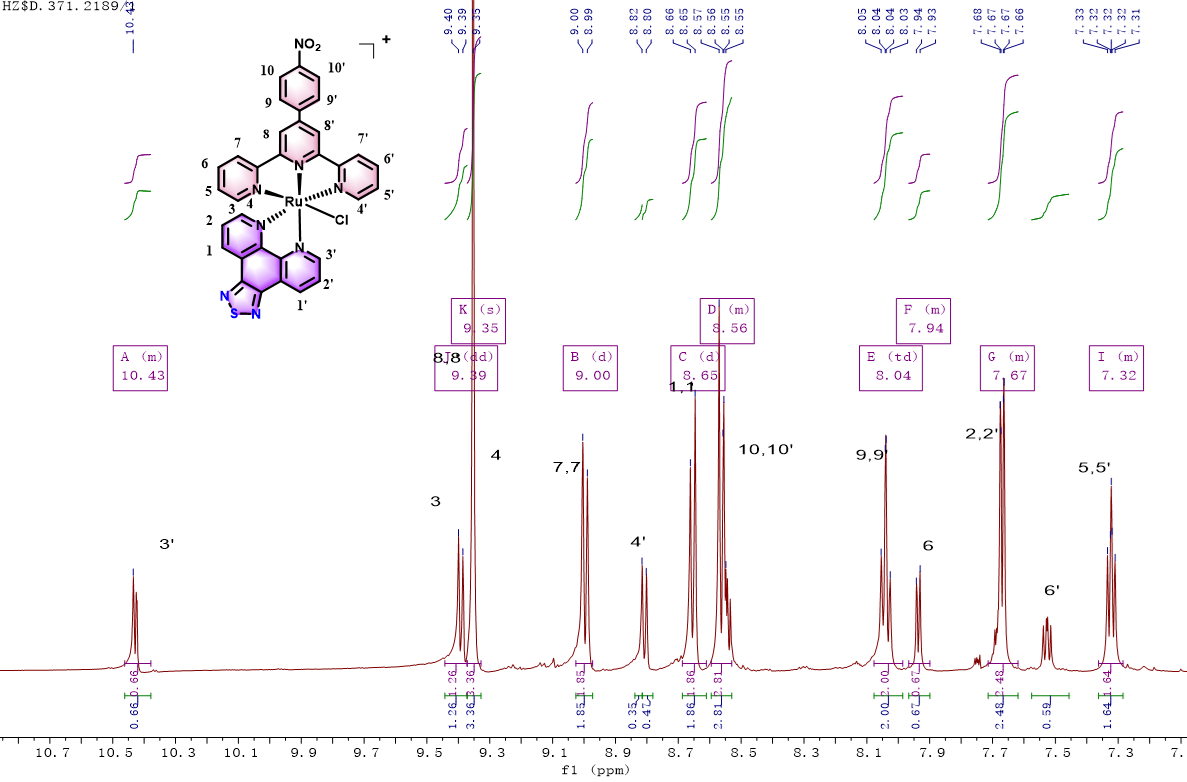
**

**Figure S4.** ^1^H NMR spectrum of **RuS** in dimethyl sulfoxide-D6.


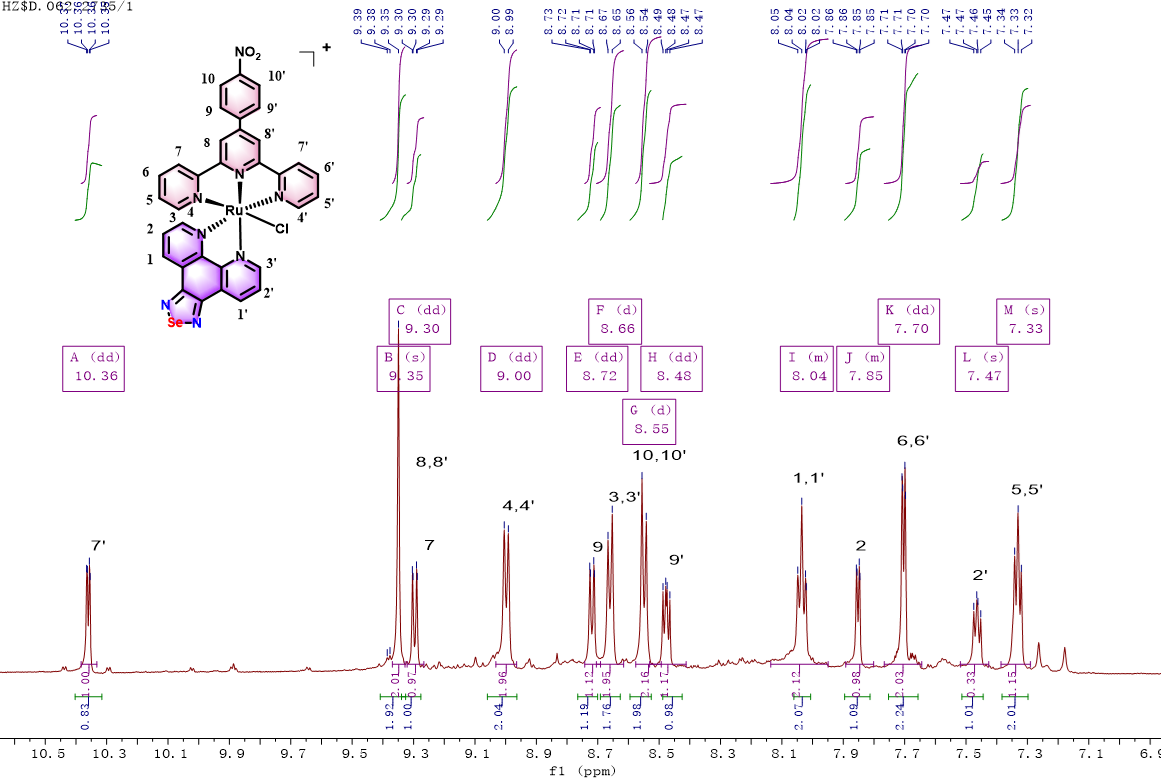


**Figure S5.** ^1^H NMR spectrum of **RuSe** in dimethyl sulfoxide-D6.


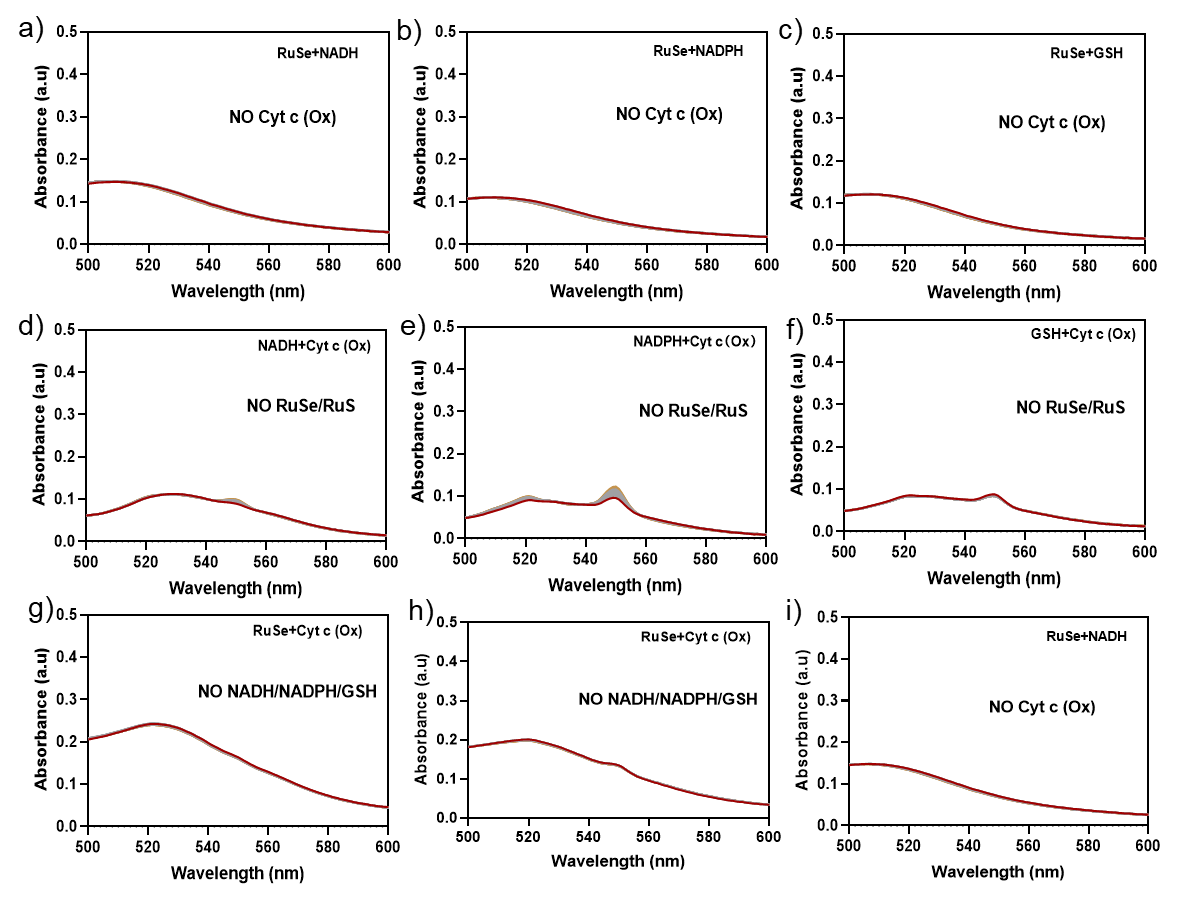


**Figure S6.** A necessary condition for complexes to intercept electrons. Absorption spectra of a) **RuSe** (20 μΜ) and NADH (100 μΜ), b) **RuSe** (20 μΜ) and NADPH (100 μΜ), c) **RuSe** (20 μΜ) and GSH (500 μΜ), d) NADH (100 μΜ) and Cyt c (Ox) (10 μΜ), e) NADPH (100 μΜ) and Cyt c (Ox) (10 μΜ), f) GSH (500 μΜ) and Cyt c (Ox) (10 μΜ), g) **RuSe** (20 μΜ) and Cyt c (Ox) (10 μΜ), h) **RuSe** (20 μΜ) and Cyt c (Ox) (10 μΜ), i) **RuSe** (20 μΜ) and NADH (100 μΜ) reacted for 2 h.

**Figure S7.** Anticancer activity of **RuSe** in HeLa cells under hypoxia (0.1% O_2_) and normal conditions (21% O_2_)（n=3）.

**
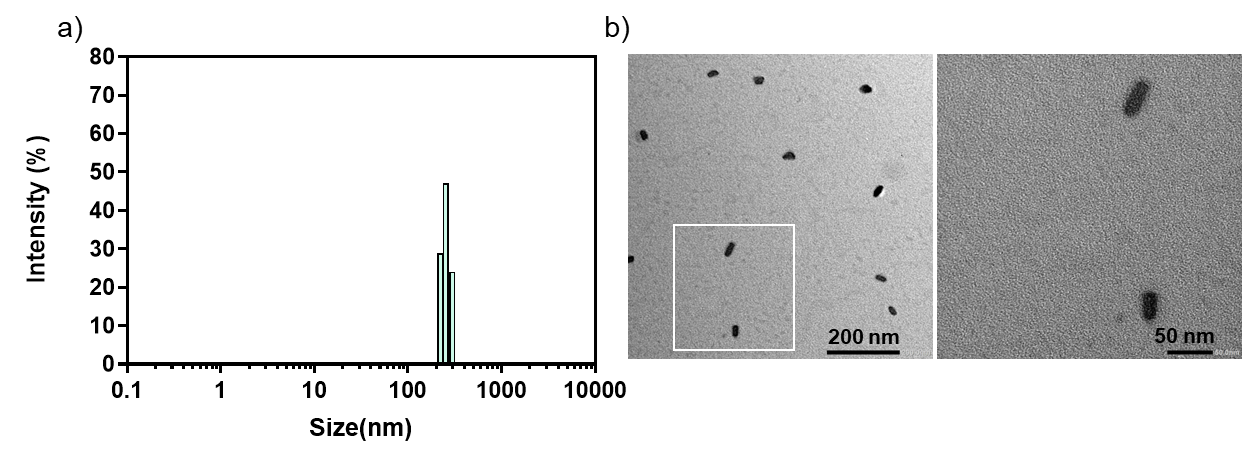
**

**Figure S8.** a) Size distribution and b) TEM characterization of **RuSeNPs**.


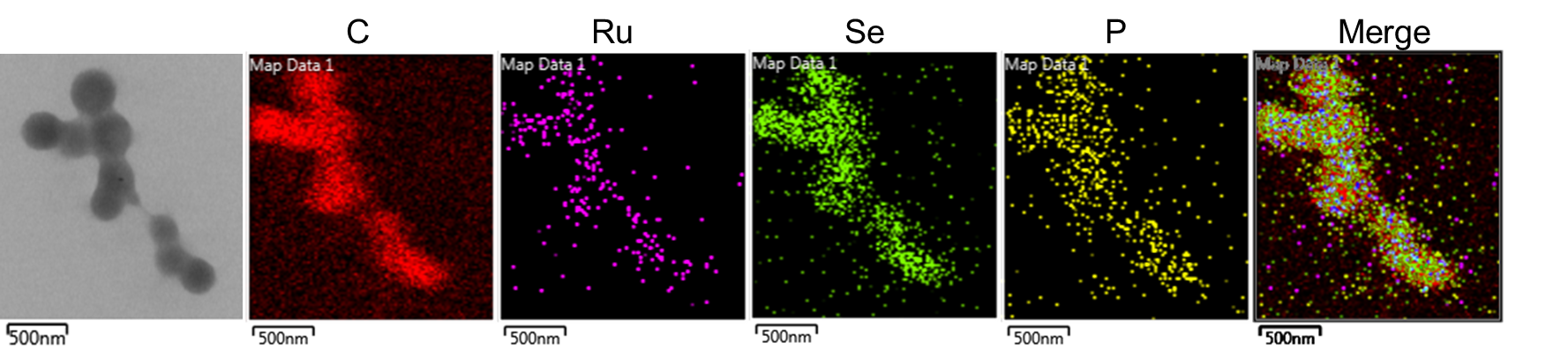


**Figure S9.** Engergy-dispersive X-ray (EDX) elemental maps of **RuSeNPs**.

**Figure S10.** Absorption spectra of **RuSeNPS** (20 μΜ), NADH (100 μΜ), and Cyt c (10 μΜ) for 2 h of reaction.

**Figure S11.** Effect of **RuSe** on hydroxyl radicals in HeLa cells.


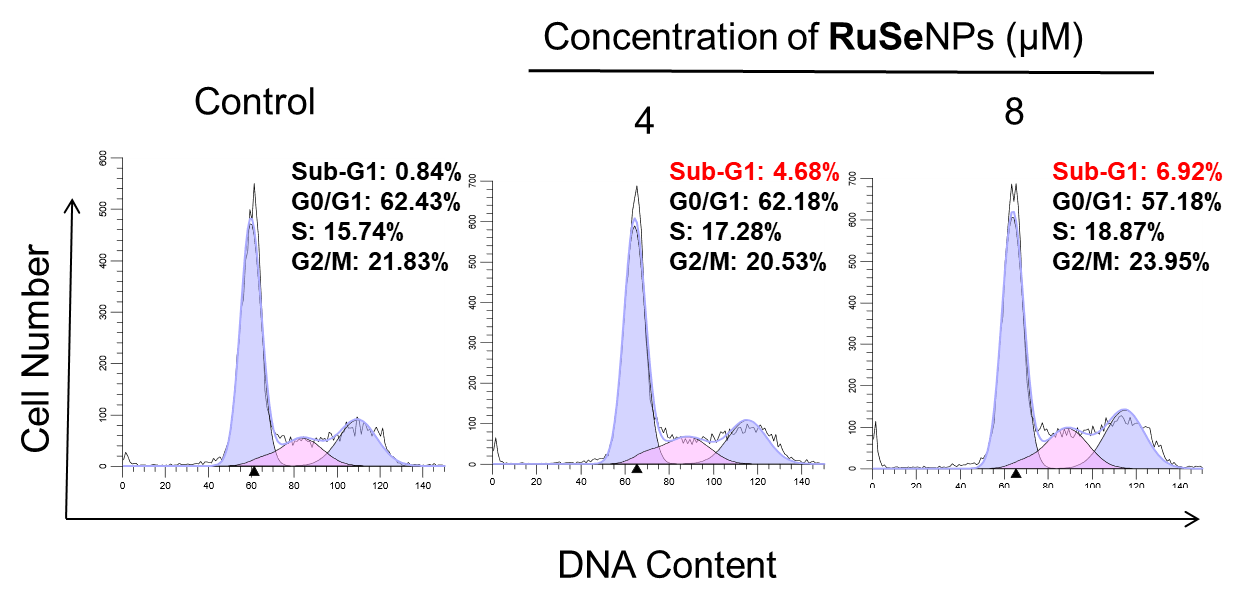


**Figure S12.** Effects of different concentrations of **RuSeNPs** on cell cycle after treatment of cells.

**3. References**

1. Z. Deng, L. Yu, et al., A selenium-containing ruthenium complex as a cancer radiosensitizer, rational design and the important role of ROS-mediated signalling *[J]. Chem Commun (Camb),* 2015, 51(13): 2637-2640.
